# Cholinergic connectivity: it's implications for psychiatric disorders

**DOI:** 10.3389/fncel.2013.00055

**Published:** 2013-05-03

**Authors:** Elizabeth Scarr, Andrew S. Gibbons, Jaclyn Neo, Madhara Udawela, Brian Dean

**Affiliations:** ^1^Department of Psychiatry, The University of MelbourneParkville, VIC, Australia; ^2^Molecular Psychiatry Laboratories, Florey Institute of Neuroscience and Mental HealthParkville, VIC, Australia; ^3^Centre for Neuroscience, The University of MelbourneParkville, VIC, Australia

**Keywords:** acetylcholine, psychiatric disorders, glutamate, GABA, dopamine, serotonin, cytokines

## Abstract

Acetylcholine has been implicated in both the pathophysiology and treatment of a number of psychiatric disorders, with most of the data related to its role and therapeutic potential focusing on schizophrenia. However, there is little thought given to the consequences of the documented changes in the cholinergic system and how they may affect the functioning of the brain. This review looks at the cholinergic system and its interactions with the intrinsic neurotransmitters glutamate and gamma-amino butyric acid as well as those with the projection neurotransmitters most implicated in the pathophysiologies of psychiatric disorders; dopamine and serotonin. In addition, with the recent focus on the role of factors normally associated with inflammation in the pathophysiologies of psychiatric disorders, links between the cholinergic system and these factors will also be examined. These interfaces are put into context, primarily for schizophrenia, by looking at the changes in each of these systems in the disorder and exploring, theoretically, whether the changes are interconnected with those seen in the cholinergic system. Thus, this review will provide a comprehensive overview of the connectivity between the cholinergic system and some of the major areas of research into the pathophysiologies of psychiatric disorders, resulting in a critical appraisal of the potential outcomes of a dysregulated central cholinergic system.

## Introduction

The central cholinergic system has been implicated in the pathophysiology of schizophrenia (Raedler et al., 2006; Scarr and Dean, 2008, 2009) as well as mood disorders (Dilsaver, 1986; Cannon et al., 2006; Gibbons et al., 2009) and is a target for drug development aimed at improving treatments for these disorders (Furey and Drevets, 2006; Freedman et al., 2008; Scarr, 2012). Whilst efforts have been made to fully understand the changes that occur in the cholinergic system with these disorders, the impact of these changes are rarely considered in the context of their effects on other systems considered pertinent to the pathophysiologies of the disorders, or conversely the influence of other systems on cholinergic functionality. Thus, this review will briefly describe the central cholinergic system and the changes reported for the cholinergic system in schizophrenia and, to a lesser extent, mood disorders. The changes in the cholinergic system will then be considered in the context of documented changes that occur in other central neurotransmitter systems in people with schizophrenia or mood disorders and how such changes may have influenced, or been influenced by, the cholinergic system. Thus, this is not a comprehensive review of either the human cholinergic system (for this see Perry et al., 1999) or of all data relating to the pathophysiologies of schizophrenia and mood disorders. Whilst the contemplations on the interactions between the human cholinergic system and other central systems are, by necessity, somewhat speculative they take as given the concept that the brain is attempting to maintain a stable environment (homeostasis) using various feedback mechanisms. Thus, this review will give a solid theoretical framework for conceptualizing the pathophysiologies of psychiatric disorders as a breakdown of complex systems rather than a single self-contained gene or biological pathway.

### The central cholinergic system

**Figure 1:**
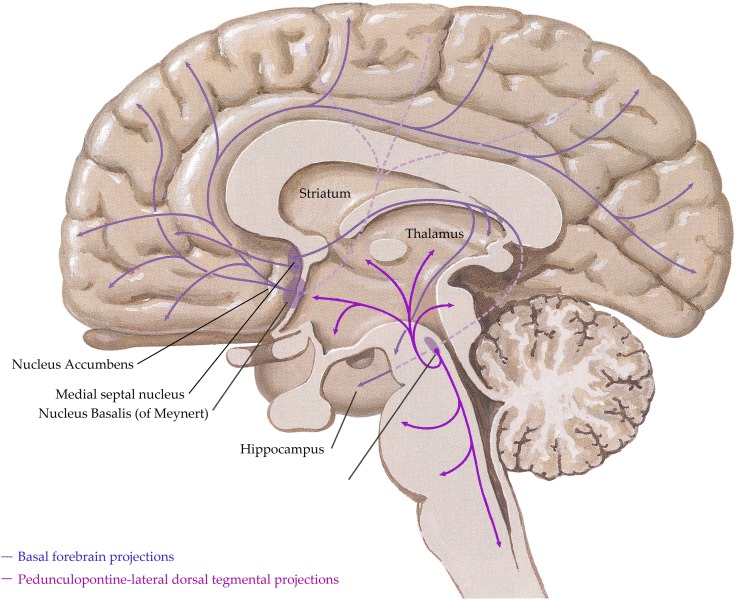
**A schematic representation of the human central cholinergic system—striatal interneurons not shown.** Adapted from (Felten and Shetty, 2010).

In the human central nervous system, the cholinergic system has evolved into a complex network with three principle components,(i) projections from nuclei of the basal forebrain; these include the medial septal nucleus, the nucleus basalis of Meynert, the vertical nucleus of the diagonal band and the horizontal limb of the diagonal band nucleus, which innervate the hippocampus, most cortical regions and some subcortical nuclei, (ii) the pedunculopontine-lateral dorsal tegmental projections from the brainstem to the thalamus, midbrain and other brainstem regions and (iii) interneurons in the striatum (most abundant) and the nucleus accumbens (Everitt and Robbins, 1997; Perry et al., 1999) (see Figure 1). Given the complex nature of the cholinergic system in the human central nervous system, it is not surprising that it controls critical, diverse functions such as sleep, cognition, motor control, and sensory processing. Importantly, all functions of the cholinergic system are controlled by the interaction of acetylcholine with two families of receptors; the nicotinic and muscarinic receptors (Dale, 1914). The nicotinic receptors are cation permeable ligand-gated ion channels, in the central nervous system the receptors consist of alpha (α1–7 and 9–10) and beta (β2–4) subunits which can be combined to form either homomeric (α7–10) or heteromeric (α2–6 and β2–4 or α7 with α9 or 10) pentameric receptors, which are named after their component subunits and appear to have distinct properties (Millar et al., 2011). By contrast, the muscarinic receptors are metabotropic, consisting of the M1–M5 receptors. M1, 3 and 5 all couple canonically to G_q/11_ proteins; stimulating hydrolysis of inositol phosphate, whilst M2 and 4 couple to G_i/o_ proteins, decreasing cyclic adenosine monophosphate(cAMP) levels. All five receptors are found in the human brain, with discreet distribution patterns, implying different functions (Challiss and Tobin, 2009). Ultimately, the functional outcome of central cholinergic stimulation depends on the balance between activation of both receptor families (Lucas-Meunier et al., 2003).

### The central cholinergic system in schizophrenia and mood disorders

The cholinergic system has been proposed to contribute to the pathophysiology of schizophrenia as a result of either an imbalance between central cholinergic and dopaminergic systems (Tandon and Greden, 1989) or an over activation of the pedunculopontine-lateral dorsal tegmental nuclei (Yeomans, 1995). More recently, it has been shown that adjunctive acetylcholinesterase inhibitors can be of use in treating visual hallucinations (Patel et al., 2010; Abad et al., 2011), suggesting a hypo-cholinergic milieu may underlie these symptoms. However, a number of trials have failed to show that cholinesterases offer any significant improvement in the symptoms of schizophrenia (Buchanan et al., 2003, 2008; Friedman, 2004; Dyer et al., 2008; Keefe et al., 2008), suggesting that the problems in the cholinergic system in schizophrenia are not simply due to changes in levels of acetylcholine.

The perturbations of the central cholinergic system have been thoroughly reviewed previously (Raedler et al., 2006; Scarr and Dean, 2008, 2009; Jones et al., 2012; Scarr, 2012) so the main points will simply be summarized:

1. The most reproduced finding is a widespread decrease in levels of muscarinic receptors in the brains of people with schizophrenia, this has been replicated in four separate postmortem collections (Mancama et al., 2003; Zavitsanou et al., 2004; Newell et al., 2007; Gibbons et al., 2013) and a neuroimaging study (Raedler et al., 2003).
2. Epibatidine binding, predominantly to the α4β2 nicotinic receptor, has been reported to be increased in people with schizophrenia (Martin-Ruiz et al., 2003).
3. The most investigated nicotinic receptor is the α7 nicotinic receptor which is associated with a sensory gating deficit present in people with schizophrenia (Adler et al., 1992) and other psychiatric disorders, although animal studies suggest that a lack of α7 receptors does not affect sensory gating (Paylor et al., 1998). In tissue from people with schizophrenia, levels of hippocampal α7 receptors have been reported to be decreased (Freedman et al., 1995) and unchanged (Thomsen et al., 2011), using a-bungarotoxin [which binds predominantly to α7 (Couturier et al., 1990)]. However, α7 mRNA expression is deceased in lymphocytes (Perl et al., 2003) and the expression of a particular splice variant is decreased in the brains from people with the disorder (Severance and Yolken, 2008), maintaining interest in this site as a potential drug target.

The first indication that the cholinergic system was involved in the pathophysiology of mood disorders came from the development of depressive symptoms in people who had been exposed to cholinesterase inhibitors (Rowntree et al., 1950; Gershon and Shaw, 1961). More recently a number of studies have implicated the muscarinic system, in particular the M2 receptor, in the mood disorders (Cannon et al., 2006; Furey and Drevets, 2006; Gibbons et al., 2009). One aspect of the pathophysiology of psychiatric disorders that is often not explored is how these changes may either arise from changes in other systems or affect the functionality of those systems. This review will explore these interactions theoretically, using data available from the literature.

## Interactions with intrinsic neurotransmitters

For the purpose of this review, the term intrinsic has been used to describe neurotransmitters that predominantly act locally throughout the central nervous system, although they may have some neurons that project across different brain regions. These neurotransmitters include the excitatory amino acid glutamate and the inhibitory amino acid gamma-amino butyric acid (GABA).

### Glutamate

#### Glutamate in the central nervous system

Glutamate is the most abundant excitatory neurotransmitter in the human central nervous system, the effects of which are mediated via two classes of receptors; ionotropic[N-methyl-D-aspartate (NMDA), 2-amino-3-(3-hydroxy-5-methyl-isoxazol-4-yl)propanoic acid (AMPA), and kainate receptors] and metabotropic (mGluR_1−8_) receptors (Traynelis et al., 2010). Like other ligand gated ion channels, the ionotropic glutamatergic receptors consist of combinations of subunits, in this instance creating tetramers, which give the receptors distinct properties. NMDA receptors are also voltage dependant and consist of two constitutive NR1 subunits, of which there are eight variants, and two NR2 subunits, of which there are four variants. AMPA receptors consist of combinations of the GluR1-4 subunits whilst the kainate receptor exists as combinations of GluR5-7 and KA1-2. Glutamate can also signal through metabotropic receptors; the Group I (mGluR_1_ and mGLUR_5_) which couple to G_q_ protein; stimulating inositol phosphate hydrolysis or Group II (mGluR_2_ and mGluR_3_) and Group III (mGluR_4_, mGluR_6_, mGluR_7_, and mGluR_8_), both of which couple to G_i_/G_o_ protein and decrease levels of cAMP (Niswender and Conn, 2010).

#### Glutamate in schizophrenia

Magnetic resonance spectroscopy studies have reported elevated glutamate levels in the hippocampus and prefrontal cortex of patients with schizophrenia (van Elst et al., 2005), highlighting these areas as major regions of glutamatergic dysfunction in the disorder. The ability of NMDA receptor antagonists, such as ketamine and phencyclidine, to induce psychotic symptoms in healthy individuals and exacerbate symptoms in people with schizophrenia (Lahti et al., 2001) led to a focus on the role of the ionotropic glutamate receptors in the pathophysiology of schizophrenia. However, the data regarding NMDA and AMPA receptor levels in schizophrenia is inconsistent (Gao et al., 2000; Dean et al., 2001; Scarr et al., 2005; Beneyto et al., 2007; McCullumsmith et al., 2007). For example, binding of [^3^H]MK-801, which binds to open NMDA receptors, in hippocampal tissue from individuals with schizophrenia has been reported to be both decreased (Beneyto et al., 2007) and unaltered (Gao et al., 2000; McCullumsmith et al., 2007). The lack of altered hippocampal gene expression (Beneyto et al., 2007) also contrasts with the report of decreased NR1 and increased NR2B subunit mRNA levels in the absence of altered [^3^H]MK-801 binding (Gao et al., 2000). NR1 protein levels are reportedly unaltered in the hippocampus (Toro and Deakin, 2005), suggesting that overall levels of the NR1 subunit are not altered. Increased expression of NR2C mRNA and an increased proportion of NR2D mRNA relative to other NR2 subunits have been reported in the prefrontal cortex from people with schizophrenia (Akbarian et al., 1996), suggesting that NDMA receptor subunit ratios may be altered in the disorder, which would impact receptor function. This possibility gains some support from the finding of increased NR1 and NR2A, but not NR2B, mRNA levels in the dorsolateral prefrontal and occipital cortices from elderly subjects with schizophrenia (Dracheva et al., 2001). However, the fact that different subunits are over expressed could either suggest that changes in NMDA receptor composition vary with age or may simply reflect the heterogeneity of the disorder.

While small decreases in AMPA receptor radio ligand binding are reported in CA2 of the hippocampus (Gao et al., 2000), other studies have failed to detect changes in hippocampal AMPA receptors (Noga and Wang, 2002; Beneyto et al., 2007). Although [^3^H]MK-801 and [^3^H]AMPA densities have generally not been altered in the prefrontal cortex in schizophrenia (Healy et al., 1998; Scarr et al., 2005), at least one study has reported increased AMPA receptor levels (Noga et al., 2001). However, this group failed to replicate their original finding in a larger cohort, reporting decreases in striatal and accumbal AMPA receptors, highlighting the heterogeneity of changes in the glutamatergic system in the disorder.

With regards to the kainate receptor, a reduction in radioligand binding density and a reduction in GluR_5_ mRNA expression have been reported in the prefrontal cortex from people with schizophrenia (Scarr et al., 2005). Whilst hippocampal kainate receptor levels are reportedly unchanged in schizophrenia (Noga and Wang, 2002; Beneyto et al., 2007), decreased GluR6 and KA2 mRNA expression has been reported in some (Porter et al., 1997) but not all (Beneyto et al., 2007) studies, suggesting that the composition of kainate receptors may also be altered in some people with the disorder.

There is increasing awareness of the potential for targeting metabotropic glutamate receptors as modulators of glutamate release, ionotropic receptor response, and glutamatergic signal transduction, in the treatment of schizophrenia (Vinson and Conn, 2012). Their prospective usefulness is supported by the report of decreased mRNA levels of the mGluR_1α_ isoform in the dorsolateral prefrontal cortex in schizophrenia (Volk et al., 2010). Although the cortical binding density of the mGluR_2_/mGluR_3_ selective ligand, [^3^H]LY354740, is reported to be unaltered in schizophrenia (Frank et al., 2011), cortical binding of [^3^H]LY341495, another mGluR_2_/mGluR_3_ selective ligand, and mGluR2 but not mGluR3 mRNA has been reported to be decreased in subjects with schizophrenia, 84% of whom died by suicide (Gonzalez-Maeso et al., 2008). LY341495 has recently been shown to be efficacious in the tail suspension test and novelty suppressed feeding test in mice (Koike et al., 2013), suggesting mGluR2/mGluR3 may be involved in mood state. Therefore, the contribution of suicide to these findings needs to be further explored. Increased hippocampal and amygdala levels of the endogenous mGluR_3_ agonist, N-acetylaspartylglutamate, have been reported in people with schizophrenia (Reynolds and Reynolds, 2011), suggesting that both ionotropic and metabotropic arms of the glutamatergic system may be affected by the disorder.

#### Cholinergic modulation of glutamatergic function

Acetylcholine has been shown to modulate glutamatergic excitatory postsynaptic potentials in several brain regions (Li and Pan, 2001; Zhang and Warren, 2002; Hamam et al., 2007), with the effects being either inhibitory or stimulatory. For example, acetylcholine has been found to increase excitatory postsynaptic potentials via nicotinic receptor signaling in the hippocampus (Radcliffe et al., 1999), hypothalamus (Li and Pan, 2001), and nucleus accumbens (Zhang and Warren, 2002). By contrast, acetylcholine or carbachol administration produce long lasting reductions of stimulus-evoked excitatory postsynaptic potential amplitude in the bed nucleus of the stria terminalis and in basal forebrain neurons (Allen et al., 2006; Guo et al., 2012), an effect supporting the finding that endogenous application of acetylcholine to hippocampal synaptosomes reduced glutamate levels (Marchi et al., 1989). The ability of the muscarinic antagonist atropine, but not nicotinic antagonists, to ameliorate these effects, combined with the ability of oxotremorine to inhibit glutamatergic currents in auditory cortical slices suggest that muscarinic receptors mediate the inhibition of glutamate release (Marchi et al., 1989; Atzori et al., 2005; Allen et al., 2006; Guo et al., 2012). Furthermore, in the nucleus accumbens, the inhibitory effects of atropine on excitatory postsynaptic potentials can be replicated with pirenzepine (Zhang and Warren, 2002), suggesting that the M1 and/or M4 are involved in regulating glutamate neurotransmission in this region. Significantly, the effect of acetylcholine on glutamatergic transmission appears to depend on the timing of the acetylcholine release relative to activating the glutamatergic neuron (Gu and Yakel, 2011). In the hippocampus, acetylcholine release prior to glutamatergic activation results in nicotinic α7 receptor-mediated long term potentiation or depression, whilst glutamatergic activation followed by acetylcholine release resulted in muscarinic receptor-mediated long term potentiation.

In the hippocampus, M1 and M3 have been shown to potentiate kainate receptor currents, increasing mossy fiber axon excitability. This modulation is subunit dependant, for example; muscarinic receptor activation potentiates heteromeric GluR6/KA1 and GluR6/KA2 receptors, but not homomeric GluR6 receptors (Benueniste et al., 2010). Thus, in schizophrenia, with reports of decreased hippocampal GluR6 and KA2 mRNA levels (Porter et al., 1997), abnormal kainate subunit ratios could affect receptor functionality. However, it is unclear whether muscarinic receptors affect signaling through kainate receptors composed of the GluR5 subunit, which is thought to underpin the reduction in cortical[^3^H]kainate density in individuals with schizophrenia (Scarr et al., 2005).

#### Glutamatergic regulation of cholinergic function

Glutamatergic signaling has been shown to modulate acetylcholine release, predominantly via the ionotropic receptors. For instance, cortical microinjections of the NMDA receptor antagonist 3-(2-Carboxypiperazin-4-yl)propyl-1-phosphonic acid (CCP) increased acetylcholine release in the nucleus accumbens, an effect blocked by local perfusions of both CCP and the AMPA receptor antagonist 6,7-Dinitroquinoxaline-2,3-dione (DNQX) (Del Arco et al., 2008). By contrast, AMPA and NMDA increase acetylcholine release in the basal forebrain (Fournier et al., 2004), where AMPA is more effective, and striatum (Anderson et al., 1994; Ishida et al., 2005), where the NMDA antagonist MK-801, but not the AMPA/kainate antagonist 2, 3-dihydroxyl-6-nitro-7-sulfamoylbenzo(f)quinoxaline (NQBX), reduced acetylcholine efflux (Anderson et al., 1994); suggesting NMDA receptors may be more potent at regulating striatal acetylcholine release. NMDA and AMPA receptors work in concert to mediate glutamatergic signaling (Maeng et al., 2008), therefore, these differences may reflect the relative contributions of the receptors in eliciting a response in different brain regions.

**Figure 2:**
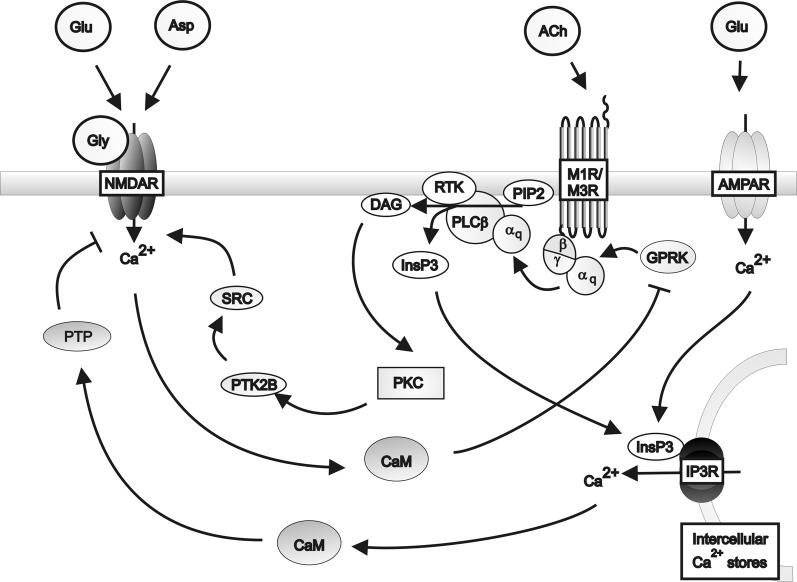
**A schematic diagram of the regulation of NMDA receptor activity by G_q_ protein-coupled muscarinic receptors in the hippocampus.** Muscarinic receptors inhibit NMDA receptor activity via the activation of protein tyrosine phosphatase mediated by inositol triphosphate receptor pathways in conjunction with AMPA receptor induced calcium release from intracellular calcium stores. Muscarinic receptors can stimulate NMDA receptor activity via the activation of Src family tyrosine kinase in response to PKC signaling. Activation of the NMDA receptor by glutamate or aspartate and the co-agonist glycine, in turn inhibits muscarinic receptor activity via calmodulin inhibition of G protein coupled receptor kinases. α_q_: G_qα_ subunit; β: Gβ subunit; γ: Gγ subunit; ACh: Acetylcholine; AMPAR; AMPA receptor; Asp: Aspartate; CaM: Calmodulin DAG: Diacyl glycerol; Glu: Glutamate; Gly: Glycine; GPRK: G protein coupled receptor kinase; InsP3: Inositol 1,4,5-trisphosphate; IP3R: Inositol triphosphate receptor; M1R: Muscarinic M1 receptor; M3R: Muscarinic M3 receptor; MEK**:** Mitogen-activated protein kinase kinase; NMDAR: NMDA receptor; PLCβ: Phospholipase C β; PIP2; Phosphatidylinositol 4,5-bisphosphate; PKC: Phosphokinase C; PTK2B: Protein tyrosine kinase 2β; PTP: Protein tyrosine phosphatase; RTK: Tyrosine kinase receptor; SRC: Src family tyrosine kinase.

The respective modulation of glutamate and acetylcholine release by cholinergic and glutamatergic pathways respectively depend on the co-expression of appropriate receptors within neurons and their synaptic connections. Microdialysis of AMPA into rat cortex facilitated acetylcholine release in the parietal and prefrontal cortices, an effect attenuated by DNQX (Nelson et al., 2005). Furthermore, DNQX partially attenuated the release of acetylcholine in the parietal cortex caused by carbachol administration to prefrontal cortex. These data suggest that cholinergic signaling in the parietal cortex is co-regulated by cholinergic and glutamatergic input from the prefrontal cortex. However, prefrontal cortical cholinergic afferents were not regulated by AMPA signaling from the parietal cortex, suggesting that the glutamatergic control is unidirectional. Further evidence for co-regulation comes from Group1 mGluRs acting in conjunction with muscarinic receptors to produce long lasting increases in excitatory postsynaptic potentials (Park and Spruston, 2012), possibly via protein kinase C (PKC)-mediated activation of Src tyrosine kinase (Lu et al., 1999). This co-regulation is supported by reports that co-administrating carbachol and rolipram, a phosphodiesterase inhibitor which prevents cAMP inhibition, produces long lasting increases in hippocampal excitatory postsynaptic potentials associated with brain derived neurotrophic factor-dependant long term potentiation (Navakkode and Korte, 2012). Further support for interactions between the two systems come from studies demonstrating that M1 receptors suppress NMDA receptor function in cornu ammonis (CA) 3 pyramidal cells (Grishin et al., 2004, 2005), by inducing tyrosine phosphatase-mediated suppression of NMDAR activity (Grishin et al., 2005) and that activation of NMDA receptor can lead to the phosphorylation and desensitization of muscarinic receptors. These data provide the basis for a proposed feedback regulatory mechanism for glutamatergic/cholinergic signaling (Butcher et al., 2009) (see Figure 2).

Studies have also shown that ventral tegmental presynaptic metabotropic glutamate and muscarinic receptors preferentially inhibit the NMDA mediated component of synaptic transmission (Zheng and Johnson, 2003). In CA1 and CA3 pyramidal cells muscarinic receptors and mGluRs can be simultaneously coupled to inhibitory and stimulatory pathways to modulate NDMAR activity in a calcium-dependent (Grishin et al., 2004), cell specific manner. Thus, these systems appear to rely on cooperation to regulate ionotropic receptor function. Hippocampal M1 and M4 are predominantly responsible for the direct cholinergic modulation of the excitatory CA1-CA3 circuit (Dasari and Gulledge, 2011). CA1 slices from mice lacking CA3 M1 have reduced mGluR mediated long term depression compared to mice with normal CA3 M1 levels (Kamsler et al., 2010), this effect was reversed by activating PKC. Together, these data led to the proposal that normal M1 levels are necessary to maintain baseline PKC activity and that additional PKC stimulation by Group 1 mGluR's facilitates mGluR-mediated long term depression at CA3 presynaptic terminals. Thus, it is possible that in schizophrenia, where deficits in M1 have been reported (Scarr et al., 2009; Gibbons et al., 2013), the PKC activity mediated by the combined signaling of M1 and mGluRs may be insufficient to maintain normal synaptic functionality.

#### Acetylcholine and glutamate in schizophrenia

The dual role of the cholinergic system, activating and inhibiting glutamatergic signaling, presents challenges in predicting the effects of (i) the M1 deficits associated with and (ii) the NMDA receptor hypofunction predicted in schizophrenia. However, animal studies have shown that inhibitory avoidance memory consolidation can be repressed by co-administration of muscarinic and NMDA antagonists to the ventral tegmentum, at doses that were ineffective when used alone (Mahmoodi et al., 2010), indicating a synergistic interaction. Thus, it is possible that the disturbances in central function seen in schizophrenia could be underpinned by a loss of synaptic plasticity due to suppression of both glutamatergic and cholinergic signaling.

Importantly, the processes governing acetylcholine and glutamate release in turn regulate and are regulated by additional neurotransmitters. For example, stimulating nicotinic receptors reduces AMPA -evoked synaptosomal dopamine overflow (Grilli et al., 2012). In addition, the co-administration of dopamine and muscarinic agonists to rat cortical slices inhibits the muscarinic receptor mediated reduction in excitatory postsynaptic potentials (Atzori et al., 2005). Therefore, the alterations in cholinergic signaling that occur in schizophrenia need to be regarded as a component of a much broader breakdown of central neurotransmission.

### Gamma-amino butyric acid

#### Gamma-amino butyric acid in the central nervous system

GABA is the major central inhibitory neurotransmitter, in mammals 25–50% of central synapses utilize GABA (Petroff and Rothman, 1998), making it essential for the balance between neuronal excitation and inhibition that underpins normal brain function (Johnston, 2005). The central effects of GABA are mediated by two receptor families, the GABA_A_ and GABA_B_ receptors (Steiger and Russek, 2004). GABA_A_ receptors are ionotropic, regulating chloride channels. The receptors are pentameric, although there are 19 different subunits within the GABA_A_ receptor family; α1–6, β1–3, γ1–3, δ, ϵ, π, ρ1–3, and θ, the minimum requirement for an active receptor are an α and β subunit (Whiting, 2003). While a GABA_C_ receptor was postulated, this receptor consists exclusively of rho (ρ) subunits and, because of their similarity to GABA_A_ subunits, is now viewed as a GABA_A_ variant (Barnard et al., 1998). GABA_B_ receptors are metabotropic, coupled to G_i/o_ proteins, and consist of 2 subunits, GABA_B1_ and GABA_B2_, both of which are necessary for functional receptors (Hyland and Cryan, 2010). As expected, given the diverse nature of the neurotransmitter, GABAergic receptors are widely distributed throughout the brain and highly expressed in cortical, hippocampal, thalamic, basal ganglia, and cerebellar structures.

#### GABA in schizophrenia

There is strong evidence to support the theory that schizophrenia is associated with deficits in GABAergic neurotransmission [see (Blum and Mann, 2002) for a detailed review]. Briefly, postmortem studies suggest that GABAergic neurons are providing insufficient inhibitory modulation in corticolimbic regions of people with schizophrenia (Benes et al., 1991, 1992, 1996b; Heckers and Konradi, 2010). Similar abnormalities have also been observed in the dorsolateral prefrontal cortex (Benes et al., 1991, 1996b) suggesting the effect could be widespread. This theory is supported by reports of pervasive increased binding densities for the GABA_A_ ligand, [^3^H]muscimol, in tissue held in a number of CNS repositories. The areas affected include the cingulate cortex, dorsolateral prefrontal cortex (Benes et al., 1996b; Dean et al., 1999a), caudate nucleus (Hanada et al., 1987), superior temporal gyrus (Deng and Huang, 2006) and hippocampus (Benes et al., 1996a) from people with schizophrenia. Further, more direct, support for the theory comes from reports of increased GABA_A_ receptor proteins in the prefrontal cortex (Ishikawa et al., 2004) of people with schizophrenia as well as increases in α1 and 5 (Impagnatiello et al., 1998) and α2 (Volk et al., 2002) subunits. The increase in GABA_A_ expression has been postulated to reflect receptor upregulation, compensating for decreased GABAergic release (Benes et al., 1996a). It is possible that the decreased activity could contribute to working memory deficits, a core cognitive problem in schizophrenia, since GABA_A_ agonists have been shown to improve performance on working memory and cognitive control tasks in people with the disorder (Lewis et al., 2008). In contrast to these increases in the GABA_A_ receptor, there have been reports of decreases in GABA_B_ receptors (Mizukami et al., 2000, 2002) and one of the subunits, GABA_B1a_, (Ishikawa et al., 2005), further implicating the neurotransmitter in the pathophysiology of the disorder and suggesting that the impact of the neurochemical balance depends upon the location and function of the GABAergic receptors.

Glutamic acid decarboxylase (GAD) 67 is essential for GABA synthesis and is used as a marker for GABAergic cells. Cortical expression of mRNA for both GAD67 and the GABA transporter, GAT1, are reported to be decreased in tissue from people with schizophrenia (Volk et al., 2000, 2001), as is cortical GAD67 protein (Curley et al., 2011). Decreased GAD67 expression has also been reported in the anterior cingulate (Woo et al., 2004, 2007) and hippocampus (Benes et al., 2007). However, two studies have reported increased cortical GAD67 mRNA and protein in people with schizophrenia (Hakak et al., 2001; Dracheva et al., 2004), suggesting that cortical dysfunction in schizophrenia is not consistently accompanied by altered expression of GAD67 mRNA. Furthermore, decreases in GAD67 occur in cortical tissue (Guidotti et al., 2000; Thompson et al., 2009) from people with bipolar disorder and cerebellum from people with mood disorders (Fatemi et al., 2005) as well as that from people with schizophrenia, raising the possibility that dysfunction of a subset of GABAergic interneurons may underpin some of the pathophysiology of major psychiatric disorders.

#### Cholinergic modulation of GABAergic function

The striatum is the major input structure of the basal ganglia and has been implicated in the pathophysiology of schizophrenia (Lester et al., 2010). GABAergic medium sized spiny projection neurons comprise more than 74% of the striatal cell population in humans (DiFiglia et al., 1976) and project almost equally to (i) nuclei that interface between the basal ganglia and the rest of the brain and (ii) other basal ganglia nuclei (Gerfen and Surmeier, 2011). These projection neurons represent the main target of the cholinergic interneurons, the predominant source of striatal acetylcholine (Izzo and Bolam, 1988; Graybiel, 1990). Although the cholinergic interneurons only constitute 1–2% of striatal cells (Graveland et al., 1985), they are vital for modulating the activity of both striatal projection neurons and GABAergic interneurons. The GABAergic interneurons make up approximately 5% of the striatal cells and are comprised of three populations, distinguishable by their expression of calcium binding proteins (Tepper et al., 2010). A striatal microcircuit has been proposed, where cholinergic interneurons communicate to one another through GABAergic interneurons (Sullivan et al., 2008), thus interactions between cholinergic and GABAergic systems would be fundamental for striatal functioning. Muscarinic receptors are thought to be expressed pre-synaptically by striatal GABAergic neurons (Grilli et al., 2009), directly inhibiting GABA release (Marchi et al., 1990; Sugita et al., 1991; Koos and Tepper, 2002). In particular, muscarine decreased GABA release (Nakamura and Jang, 2012), possibly by activating pre-synaptic M4 receptors. Investigations in the amygdala, nucleus accumbens and striatum confirmed that acetylcholine and muscarine inhibit GABA release, an effect attenuated by pirenzepine, an M1/M4 antagonist (Sugita et al., 1991).

Nicotinic receptors, on the other hand, appear to facilitate GABA release (Lena et al., 1993; Wonnacott et al., 2006). For example, nicotine increased the frequency, but not amplitude of spontaneous inhibitory post-synaptic potentials of hippocampal neurons (Fisher et al., 1998). It was also shown to increase the amplitude of evoked inhibitory post-synaptic potentials (Radcliffe et al., 1999). This effect may account for the activation of choline acetyl transferase expressing neurons in the nucleus accumbens increasing the frequency of GABA_A_ –mediated inhibitory post-synaptic potentials (Witten et al., 2010). However, the nicotinic mediated release of GABA was prevented by activation of M4 receptors (Grilli et al., 2009), suggesting that both muscarinic and nicotinic receptors may coexist on GABAergic terminals and that the impact of nicotinic receptors on GABA release can be modulated by muscarinic receptors. Finally, studies have reported that the nicotinic effect appears to be indirect, involving either dopamine (Kayadjanian et al., 1994) or serotonin (Bianchi et al., 1995) as the intermediary. Together, these data indicate that the consequence of acetylcholine will depend on the relative distribution of muscarinic and nicotinic receptors and that the effects may be mediated by a second system.

#### GABAergic regulation of cholinergic function

To obtain insight into GABA-acetylcholine interactions, a number of studies investigated the effects of GABA agonists, such as; muscimol, progabide, SL75102, δ-aminovaleric acid, and 2-pyrrolidone, on acetylcholine levels. In a number of brain regions, low doses of GABA agonists increased acetylcholine levels (Scatton and Bartholini, 1982), probably via stimulation of GABA_A_receptors located on cholinergic cells. Earlier studies had suggested that the action of GABA was indirect, with dopamine suggested as an intermediary (Ladinsky et al., 1976; Javoy et al., 1977). However, lesions of the dopaminergic and serotonergic pathways did not affect GABA mediated responses (Scatton and Bartholini, 1982), indicating that they could play a minor role. The same study found that lesions of the glutamatergic cortico-striatal projections ablated the GABAergic inhibition of cholinergic transmission (Scatton and Bartholini, 1982), indicating that GABA may indirectly modulate acetylcholine release by inhibiting the excitatory input to the cholinergic interneurons. Together, these studies illustrate the complexity of interactions between the cholinergic and GABAergic systems, which could affect a diverse set of central functions, including cognitive processes which may be relevant to schizophrenia (Lewis et al., 2008).

#### Acetylcholine and GABA in schizophrenia

The number of striatal cholinergic interneurons has been shown to be decreased in people with schizophrenia (Holt et al., 1999), this could disrupt the normal function of GABAergic projection neurons thereby contributing to the prefrontal cortical dysfunction associated with schizophrenia. With respect to the neurochemical changes associated with schizophrenia, the widely replicated increase in binding to the GABA_A_ receptors (Benes et al., 1996b; Dean et al., 1999a; Deng and Huang, 2006) would be expected to result in a reduced cholinergic activity. This, in turn, should lead to increased levels of post-synaptic cholinergic receptors in an attempt to compensate for transmission deficit as well as potentially causing a decrease in pre-synaptic receptors to reduce the feedback regulation of the cholinergic system. These outcomes are not in keeping with the alterations in the cholinergic system commonly reported in schizophrenia [see “The Central Cholinergic System in Schizophrenia and Mood Disorders” and (Scarr and Dean, 2009)]. However, given the modulation of the GABAergic system by nicotinic receptors, the decreased expression of some nicotinic α7 receptor variants (Severance and Yolken, 2008), may reduce GABA release (Lena et al., 1993; Wonnacott et al., 2006), resulting in increased levels of postsynaptic GABAergic receptors, an effect widely reported in schizophrenia (Benes et al., 1996b; Dean et al., 1999a; Deng and Huang, 2006). Whilst this concept appears to have face validity, it will depend on whether the α7 receptor does indeed modulate GABA and should also result in changes in GABA_B_ receptors, which have been reported to be decreased in the hippocampus (Mizukami et al., 2000) and the entorhinal cortex (Mizukami et al., 2002) as have cortical GABA_B1a_ subunits (Ishikawa et al., 2005). Since GABA_B_ receptors have been shown to be both pre- and post-synaptic (Bettler et al., 2012), it is possible these decreases reflect an attempt to reduce the feedback on the pre-synaptic neuron. However, until the localization of the reduced GABA_B_receptors is known, this association between nicotinic and GABAergic systems in schizophrenia remains speculative.

## Interactions with other projection systems

The systems considered in this section are neurotransmitter systems whose neurons arise from discreet brain structures and project to distal regions of the brain, affecting the activity of the intrinsic neurotransmitters in those regions. The choice of projection systems to be included in this review was driven, in part, by the known pathophysiologies of schizophrenia, and therefore focuses on the dopaminergic and serotonergic systems.

### Dopamine

#### Central dopaminergic systems

**Figure 3:**
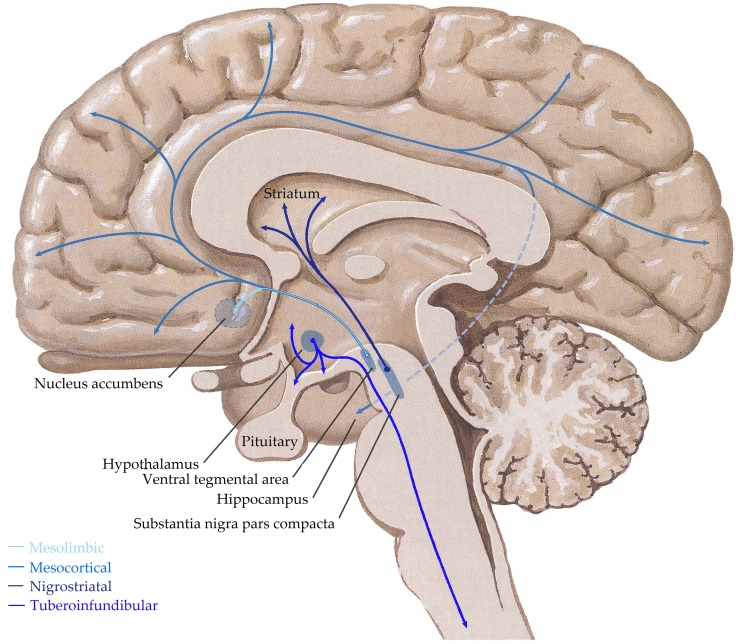
**Schematic representation of the human central dopaminergic systems.** Adapted from (Felten and Shetty, 2010).

Dopaminergic cells are found almost exclusively in the substantia nigra (SN) and ventral tegmental area (VTA), forming four major dopaminergic pathways in the mammalian brain, these are the (i) mesolimbic, (ii) mesocortical, (iii) nigrostriatal, and (iv) tuberoinfundibular pathways (Albanese et al., 1986) (see Figure 3). In brief, the mesolimbic pathway consists of dopamine-containing cell bodies in the VTA, which project to limbic structures such as the nucleus acumbens, hippocampus, and amygdale as well as the medial prefrontal cortex (Albanese and Minciacchi, 1983). This pathway is thought to be important for the acquisition of behaviors reinforceable by the inappropriate stimuli of addictive drugs (Le Moal and Simon, 1991; Lester et al., 2010). The mesocortical system is closely associated with the mesolimbic system, connecting the VTA to the cerebral cortex, particularly the frontal cortex. It is considered essential for cognitive functions involving the dorsolateral prefrontal cortex and is thought to play a major role in memory, motivation, and emotional response (Noback et al., 2005). Dopamine-containing cell bodies originating in substantia nigra pars compacta (SNpc) of the midbrain and projecting predominantly to the caudate-putamen constitute the nigrostriatal pathway (Albanese et al., 1986), which is thought to play a major role in motor coordination and has been implicated in Parkinson's disease and chorea. Finally, the tuberoinfundibular pathway originates in the arcuate and periventricular nuclei of the hypothalamus and projects to the median eminence, the infundibular and the pituitary (Albanese et al., 1986); where it inhibits prolactin secretion.

There are two types of G-protein coupled dopamine receptors, which are widely distributed centrally; D1-like receptors (D1 and D5), which couple to G_s_ proteins and stimulate cAMP production and D2-like receptors (D2,3, and 4), which couple to G_i/o_ proteins and either have no effect on or inhibit cAMP (Schetz, 2009). D1and D2 receptors are widespread throughout the central nervous system and are generally present at higher levels than the D3, 4, and 5 receptors; such a distribution is in keeping with the diverse functions these receptors are implicated in mediating (Mansour and Watson, 1995).

In both Lewy Body dementia and Alzheimer's disease, where there is a loss of cholinergic neurons, patients have a loss of cognitive function and neuropsychiatric symptoms. Although both groups have similar levels of delusions, anxiety, and depression, patients with mild Lewy Body dementia have more visual and auditory hallucinations than patients with Alzheimer's disease (Auning et al., 2011; Bjoerke-Bertheussen et al., 2012). This difference in clinical presentation may be due to the increased severity in cholinergic degeneration seen in Lewy Body dementia (Francis and Perry, 2007) or to the dopaminergic degeneration that also occurs in this disorder (Klein et al., 2010). Thus, the benefits of understanding the interactions between the cholinergic and dopaminergic systems will be beneficial for disorders other than schizophrenia and mood disorders.

#### Dopamine in schizophrenia

The dopaminergic system has long been considered a major component of schizophrenia pathophysiology (Carlsson, 1988). The dopamine hypothesis of schizophrenia is based on the observation that stimulation of the dopaminergic system with drugs such as amphetamine often leads to transient psychotic symptoms, and that a large number of antipsychotics used to treat the disorder block the activity of dopamine receptors (see Carlsson et al., 1997; Emilien et al., 1999). Although it has long been accepted that glutamate and GABA modulate activity of dopamine neurons, the discovery that acetylcholine may be as important in controlling dopamine release was made more recently. It is now postulated that an imbalance between dopaminergic and cholinergic systems contribute to disorders of the central nervous system (Tandon and Greden, 1989). Therefore, restoring the balance between the two systems is considered a practical treatment strategy (Knable and Weinberger, 1997).

The classic hypothesis for schizophrenia proposed that hyperactivity of dopaminergic transmission was responsible for the positive symptoms, however, the awareness of enduring negative symptoms and cognitive deficits, with their resistance to D2 antagonism, led to a reformulation of this hypothesis. Functional imaging studies suggested that altered functionality of the prefrontal cortex [PFC; see (Knable and Weinberger, 1997)] may contribute to the symptomatology of schizophrenia. Numerous pre-clinical studies have demonstrated the importance of prefrontal activation of D1 receptors for optimal PFC performance, [see (Goldman-Rakic et al., 2000) for example]. These findings led to the current view that an imbalance between subcortical and cortical dopaminergic systems is responsible for the symptoms of schizophrenia; a hyperactivity of the dopaminergic system in the subcortical regions (resulting in hyperstimulation of D2 receptors) causes the positive symptoms while hypoactivity of the mesocortical dopamine projections (resulting in hypostimulation of D1 receptors) is responsible for both negative symptoms and cognitive impairment (Guillin et al., 2007). In support of this hypothesis, imaging studies have consistently demonstrated that schizophrenia is associated with increased presynaptic activity of dopaminergic neurons projecting to the striatum, and a decrease in D1 receptor-like binding, as measured with positron emission tomography, was reported in the PFC of patients with schizophrenia, correlating with cognitive dysfunction and negative symptoms (Okubo et al., 1997). This correlation with symptoms has consistently been reported, even though the decrease in binding was not always replicated, with reports of increased (Abi-Dargham et al., 2002) and unchanged (Karlsson et al., 2002) levels of D1 receptors.

Blocking the D2 receptor reduces positive symptoms in people with schizophrenia (Carlsson, 1974; Creese et al., 1976; Seeman et al., 1976; Kapur and Remington, 2001). However, the data from studies on the levels of D2-like receptors are highly variable, with reports of increases (Lee et al., 1978; Mackay et al., 1982), decreases (Dean et al., 2004) and no change (Reynolds et al., 1981). To further complicate matters, the changes appear to be region specific (Dean et al., 2004) and it is possible that antipsychotic drugs may affect the outcomes (Mackay et al., 1980), although there is debate about this point (Mita et al., 1986). D4 receptors have consistently been reported to be increased (Seeman et al., 1993; Sumiyoshi et al., 1995; Marzella et al., 1997), whilst there is little data available for D3 receptors many ligands see D2/D3 receptors, hence the reporting of D2-like receptors.

The apparent inconsistencies between dopaminergic systems has been resolved by studies showing reciprocal and opposite regulation between the cortical and subcortical systems (Pycock et al., 1980) [for review see (Tzschentke, 2001)], with prefrontal dopaminergic activity exerting an inhibitory influence on subcortical dopaminergic activity (Deutch et al., 1990; Kolachana et al., 1995; Karreman and Moghaddam, 1996; Wilkinson, 1997). Significantly, chronic blockade of D2 receptors leads to a decrease in D1 receptors in the PFC region, along with impairments in working memory in non-human primates (Castner et al., 2000). Thus, there is evidence that a dopaminergic imbalance may be involved in schizophrenia, contributing to some of the key symptom domains associated with the disorder.

#### Cholinergic regulation of dopaminergic function

The striatum is densely innervated by tonically active cholinergic interneurons (Butcher and Woolf, 1984; Woolf, 1991; Aosaki et al., 1995; Bennett and Wilson, 1999), which interact closely with dopaminergic neurons to modulate their activity. Given the heterogeneity of muscarinic receptors and their signaling cascades, it is not surprising that activating muscarinic receptors results in both excitation and inhibition of dopaminergic activity in the basal ganglia. There is considerable evidence that interactions between cholinergic and dopaminergic systems are critical for the proper regulation of motor control, a function strongly attributed to the striatum. For example, an imbalance between striatal muscarinic and dopaminergic tone is thought to contribute to the severe motor deficits experienced by people with Parkinson's disease and other extrapyramidal motor disorders (Hornykiewicz, 1981; Brown and Taylor, 1996). Indeed, dopamine agonists and muscarinic antagonists are useful in the treatment of Parkinson's disease (Hornykiewicz, 1981; Fahn et al., 1990; Brown and Taylor, 1996), where degeneration of dopaminergic neurons in the SNpc causes reduced striatal dopaminergic function (Hornykiewicz, 1981; Graybiel, 1990).

All muscarinic receptors are expressed in the striatum, suggesting all have the potential to modulate dopamine release (Weiner et al., 1990; Bernard et al., 1992; Yasuda et al., 1993; Hersch et al., 1994). Almost all D1 receptor-expressing striato-nigral neurons also express both M1 (Weiner et al., 1990; Bernard et al., 1992) and M4, whereas the D2 receptor expressing striatal-palladial neurons express M1 but less than half express M4. Pharmacological determinations of which muscarinic receptors modulated cholinergic and dopaminergic interactions were hindered by a lack of specific ligands, resulting in disparate findings (Raiteri et al., 1984; Schoffelmeer et al., 1986; De Klippel et al., 1993; Smolders et al., 1997). The development of more specific ligands revealed that stimulating M1/M4 receptors causes potent dopamine release in the striatum and cortex (Bymaster et al., 1994; Ichikawa et al., 2002; Goldman-Rakic et al., 2004). Furthermore, the cognitive deficits produced by scopolamine, a muscarinic antagonist, could be reversed by D1 blockade (McGurk et al., 1988).

A more direct approach to delineating the muscarinic-dopaminergic interactions, came from studies on M1-5 receptor deficient mice (Hamilton et al., 1997; Gomeza et al., 1999a,b; Matsui et al., 2000; Miyakawa et al., 2001; Yamada et al., 2001a,b; Fisahn et al., 2002). In striatal slices, a lack of M1 or M2 receptors did not affect oxotremorine-mediated dopamine release (Zhang et al., 2002). However, *in vivo* microdialysis showed that M1-deficient mice had elevated striatal extracellular dopamine (Gerber et al., 2001), possibly due to extrastriatal receptors exerting an inhibitory striato-nigral feedback. Further studies found that M2 were required for muscarinic regulation of dopamine release in dorsal but not limbic striatal regions (Threlfell et al., 2010) and that oxotremorine-mediated dopamine release was enhanced in M3 KO mice and abolished in M4 KO mice (Zhang et al., 2002), suggesting that M3 receptors inhibit and M4 receptors promote striatal dopamine output. Furthermore, blockade of M3 receptors increased striatal but not nucleus accumbens dopamine efflux, suggesting that muscarinic modulation of dopaminergic transmission is region specific (Miller and Blaha, 2005). In addition, M4 receptors appear to inhibit dopamine D1 receptor-stimulated adenylyl cyclase activity (Olianas and Onali, 1996; Olianas et al., 1996), which would account for the hypersensitivity of mice lacking M4 receptors to the stimulatory locomotor effects of D1 receptor activation (Gomeza et al., 1999b), possibly due to a lack of striatal inhibition. Finally, M5 are the only muscarinic receptors expressed on dopaminergic neurons in the substantia nigra pars compacta (Weiner et al., 1990), where they regulate dopamine release (Forster et al., 2002; Yamada et al., 2003; Bendor et al., 2010; Steidl et al., 2011). Deletion of theM5 results in impaired dopamine release (Yamada et al., 2001a), improved latent inhibition (Wang et al., 2004) and increased D2 expression in the striatum, hypothalamus, hindbrain, and tectum (Zhang et al., 2002), possibly reflecting a compensatory mechanism. This is of interest because striatal D2 receptors have been shown to be upregulated in schizophrenia (Laruelle et al., 1996; Abi-Dargham et al., 1998) and unmedicated patients with acute schizophrenia display poor latent inhibition (Gray et al., 1995), thus M5 dysfunction might occur in schizophrenia.

The initial association between nicotine addiction and dopaminergic striatal signaling suggested the existence of a nicotinic-dopaminergic interaction (see Corrigall, 1999, for a review). Studies showed that dopaminergic antagonists, lesions of dopaminergic neurons or of the nucleus accumbens (Corrigall et al., 1992) could reduce nicotine self-administration. Nicotinic receptors are commonly expressed pre-synaptically, with activation resulting in rapid increases in neurotransmission. This, coupled with the overlap of the striatal cholinergic and dopaminergic systems, suggests that frequent, rapid regulation occurs between the two (Zhou et al., 2001).

Systemic nicotine has been shown to increase dopamine release in the mesolimbic (Imperato et al., 1986; Damsma et al., 1989; Benwell and Balfour, 1994; Nisell et al., 1994a; Pontieri et al., 1996), nigrostriatal (Benwell and Balfour, 1994; Imperato et al., 1986; Toth et al., 1992), and mesocortical (Toth et al., 1992; Nisell et al., 1994a) systems. Microdialysis experiments showed nicotine, applied to cortical terminal regions, evokes an increase in extracellular dopamine levels, albeit to a lesser extent than in the striatum and accumbens (Mifsud et al., 1989; Nakamura et al., 1992; Toth et al., 1992; Nisell et al., 1994b; Marshall et al., 1997), possibly due to fewer nicotinic receptors on cortical dopaminergic terminals. Blockade of nicotinic receptors in the VTA abolished the nicotine-induced increase in dopamine and its metabolites, however blockade in the nucleus acumbens had no effect (Nisell et al., 1994b), suggesting nicotine was acting via somatodendritic receptors on dopamine neurons, i.e., pre-synaptic.

Subsequent experiments demonstrated that striatal nicotinic control of dopamine release is mediated predominantly by receptors containing the β2 subunit (Zhou et al., 2001), a finding supported by a report that α4β2 agonists stimulate dopamine and acetylcholine release in the hippocampus and frontal cortex in rats (Bontempi et al., 2001). There is also evidence that the roles of α7 and α4β2 receptors in the cognitive impairments associated with schizophrenia are mediated through the dopaminergic system. For example, haloperidol potentiated the memory deficits induced by a nicotinic antagonist. The deficit was potentiated by the D2 antagonist raclopride, but not the D1 antagonist SCH 23390 (McGurk et al., 1989), and reversed by D2 but not D1 agonists (Levin et al., 1989), suggesting this effect is due to D2 blockade. Furthermore, a combination of an acetylcholinesterase inhibitor and risperidone produced synergistic improvements of cognitive impairment and increased extracellular dopamine in the mouse prefrontal cortex. These effects were blocked by D1 and nicotinic antagonists but not a muscarinic antagonist (Wang et al., 2007), indicating the effect is independent of the muscarinic system, and that a combination of nicotinic and D1 agonism may improve cognition in people with schizophrenia, possibly via activation-dependent effects on the D1 receptor in the prefrontal cortex. Nicotine can improve attention and some aspects of positive symptoms in schizophrenia (Nisell et al., 1995), thus, it has been postulated that the higher rate of smoking observed in patients with schizophrenia may be a form of self-medication; enhancing cortical dopamine release.

Whilst it is apparent that nicotine can stimulate dopamine release (Marshall et al., 1997), the mechanism is complex, with glutamatergic transmission involved in nicotine-induced dopamine release from the striatum (Toth et al., 1992; Marshall et al., 1997). Local applications of NMDA antagonists significantly reduced the effect of nicotine (Toth et al., 1992). It was also demonstrated that nicotine elevated striatal glutamate, an effect blocked by a nicotinic antagonist (Toth et al., 1992). Together, these data suggest that presynaptic nicotinic receptors, on glutamatergic terminals, stimulate glutamate release which in turn acts on NMDA receptors on dopaminergic terminals to increase dopamine release (Toth et al., 1992; Marshall et al., 1997).

#### Dopaminergic regulation of cholinergic function

Early studies proposed that dopamine inhibits acetylcholine transmission, with the development of more specific ligands and newer techniques, it is now apparent that blockade of D1 receptors reduces acetylcholine release whilst activation stimulates release (Bertorelli and Consolo, 1990; Damsma et al., 1990; Consolo et al., 1992; Di Chiara et al., 1993). Conversely, activation of D2 receptors reduces acetylcholine release while inhibition of these receptors stimulates the release (Damsma et al., 1991). Studies have revealed a polymorphism in the untranslated region of the D1 receptor to be associated with nicotine dependence (Huang et al., 2008), alcohol dependence (Batel et al., 2008) and autism spectrum disorder (Hettinger et al., 2008). Interestingly, differential expression of the alleles was affected by a microRNA, miR-504 (Huang and Li, 2009), suggesting that as we discover more about the processes involved in the regulation of gene product expression our understanding of the mechanisms contributing to the pathophysiology of psychiatric disorders will also be expanded.

#### Acetylcholine and dopamine in schizophrenia

The lack of consensus regarding the status of dopaminergic receptors in schizophrenia makes it difficult to speculate as to whether they may impact on cholinergic function. Conversely, there is strong evidence to suggest that nicotine stimulates dopamine release, with receptors containing a β2 subunit playing a significant role (Zhou et al., 2001). Thus, given the reported increase of such receptors in schizophrenia (Martin-Ruiz et al., 2003), it is possible that one consequence is a facilitation of dopamine release, either directly or indirectly (Toth et al., 1992; Marshall et al., 1997). With respect to the muscarinic system, M1 receptors are consistently reported to be decreased in the brains from people with schizophrenia. Since M1 null mice have increased levels of striatal dopamine, it is possible that the low levels of M1 also contribute to an increased dopamine release. Finally, there is a single report of hippocampal M4 receptors being decreased in tissue from people with schizophrenia (Scarr et al., 2007), mice that lack M4 receptors appear to be hypersensitive to D1 stimulation. Unfortunately, given the disparity of data related to D1 receptors in schizophrenia, it is not clear whether the decrease in M4 receptors contributes to the imbalance of dopaminergic systems postulated to exist in schizophrenia. Therefore, it is possible, at this stage, to suggest that the changes in nicotinic and M1 receptors may play a role in the dopaminergic dysregulation. However, given the possibility that the glutamatergic system acts as an intermediary for some, if not all of the cholinergic regulation of dopamine function, we need to consider schizophrenia as a disorder of central neurotransmission, rather than focusing on particular combinations of neurotransmitters.

### Cholinergic interactions with serotonin

#### Central serotonergic systems

**Figure 4:**
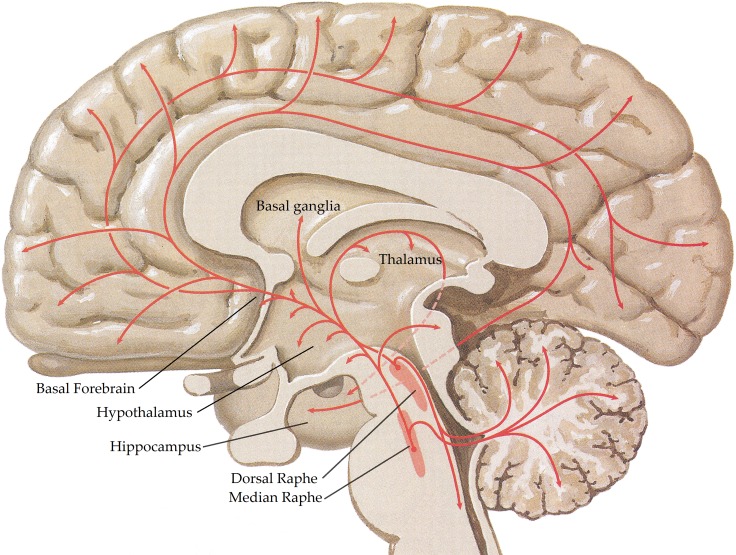
**Schematic representation of the human central serotonergic systems.** Adapted from (Felten and Shetty, 2010).

The central serotonergic system is widespread, innervating nearly all brain regions [see (Hornung, 2003) for review; Figure 4] and exerting its actions via 11 functional serotonergic receptors (Sharman et al., 2011). The majority of projections arise from the dorsal and median raphe nuclei, in the brainstem (Olszewski and Baxter, 1954), which innervate the amygdala, basal forebrain, hypothalamus, thalamus, caudate-putamen, cerebral cortex, and part of the hippocampus (Azmitia and Segal, 1978; Steinbusch, 1981). Axons of the dorsal raphe innervate all of the cerebral cortex with more dense innervation in the primary sensory areas (Wilson and Molliver, 1991) and, due to the lack of classic synapses, are thought to be involved in diffuse volume transmission rather than the targeted transmission associated with the axons from the median raphe, terminals of which are most abundant in the frontal cortex and hippocampus (Hornung, 2003). Whilst many structures are innervated by both dorsal and median raphe nuclei, the hippocampus receives predominantly median inputs whilst the thalamus, caudate, and putamen are heavily innervated by the dorsal raphe (Geyer et al., 1976).

The large family of serotonergic receptors gives the neurotransmitter an even greater functional capacity than is conferred by the diffuse serotonergic projections. In this review 5-ht_5a_, and 5-ht_1e_ receptors are not considered because of their lack of a robust signal in native tissue. Of the remaining 11 receptors, most are metabotropic; 5-HT_4, 6&7_ receptors canonically couple to G_s_, increasing levels of cAMP; the 5-HT_1_ receptors canonically couple with G_i/o_ and reduce levels of cAMP whilst the 5-HT_2_ receptors canonically couple to G_q/11_ and increase inositol phosphate hydrolysis. The 5-HT_3_ receptor is a pentameric ligand-gated cation channel; 5-HT_3A_ subunits can form functional homomeric receptors whilst the 5-HT_3B, C, D &E_ subunits form functional heteromeric receptors with 5-HT_3A_ subunits (Barnes et al., 2012). The diversity of the central serotonergic system means it regulates a range of processes including cognition and emotion (Buhot et al., 2000), as well as being implicated in the pathophysiologies of central nervous system disorders, particularly schizophrenia [see (Maris, 2002; Ohtsuki et al., 2002; Tanaka et al., 2003) for example].

#### Serotonin and schizophrenia

The first suggestion that serotonin might play a role in the pathophysiology of schizophrenia arose from the observation that lysergic acid diethylamide (LSD), a serotonergic agonist, caused psychoses which were proposed to have similarities to the positive symptoms of schizophrenia (Wooley and Shaw, 1954). After initial interest, the role of serotonin was largely unexplored until the advent of the second generation of antipsychotic drugs, with their high affinities for various serotonergic receptors, in particular as antagonists at the 5-HT_2A_ receptor (Meltzer, 1995). Although M-100907, a selective 5-HT_2A_ antagonist, failed to show an antipsychotic effect in phase III clinical trials (de Paulis, 2001) interest in the role of central serotonin in schizophrenia continues.

A number of studies looked at the major serotonin metabolite, 5-hydroxyindoleacetic acid, in cerebrospinal fluid from people with schizophrenia; the results are inconclusive, with reports of increases, decreases, and no change (see Abi-Dargham et al., 1997). Due to the rapid degradation of neurotransmitters and their metabolites, most studies have focused on markers of the serotonergic system as indirect indices of serotonergic function. In brief, the strongest indication that serotonin plays a role in the pathophysiology of schizophrenia is the widespread decreases reported in the 5-HT_2A_ receptor by multiple studies [see (Dean, 2003) for a comprehensive review]. Although a recent study reported increased cortical levels of 5-HT_2A_ receptors in the cortex of people with schizophrenia (Gonzalez-Maeso et al., 2008), a confound that was not adequately discussed was that 21 of the 25 subjects with schizophrenia had died by suicide compared to a 0% suicide rate in the control subjects. This is important because a number of studies have reported increased levels of central 5-HT_2A_ receptors in people who died as a result of suicide (see Stanley and Mann, 1983; Hrdina and Du, 2001; Pandey et al., 2002; Garbett et al., 2008; Klempan et al., 2009; for example). Therefore, it is not possible to determine whether the findings in this study relate to mode of death or the pathophysiology of schizophrenia. Given that the cholinergic and serotonergic systems are both implicated in the pathophysiology of schizophrenia and the considerable overlap between the two systems, it remains to be determined whether the changes in these systems are linked or whether they occur independently of each other.

#### Cholinergic regulation of serotonin

Projections from the medial septal nucleus and the diagonal band of Broca innervate the raphe nuclei (Kalén and Wiklund, 1989) in the rat, suggesting that the cholinergic system exerts a regulatory influence over the serotonergic network. However, a lack of [3H]choline in the basal forebrain nuclei following loading of the raphe nuclei and a lack of colocalization of the retrograde tracer with acetylcholinesterase immune reactivity in the basal forebrain lead the authors to suggest that few of the neurons projecting to the raphe were cholinergic. Later studies mapping afferents of the raphe nuclei did not identify the phenotypes of cells in the basal forebrain (Peyron et al., 1997). Conversely, a study mapping the projections of the pedunculopontine and laterodorsal tegmental nuclei of the brainstem found that cells positive for choline acetyltransferase projected to all of the raphe nuclei (Woolf and Butcher, 1989). These projections, coupled with the presence of nicotinic receptors on serotonergic neurons in the dorsal raphe (Cucchiaro et al., 2005; Galindo-Charles et al., 2008), suggest acetylcholine modulates the serotonergic system. Particularly since nicotine has been shown to increase serotonin release (Ma et al., 2005) and the firing rate of some serotonergic neurons in the dorsal raphe (Chang et al., 2011). However, it was previously shown that acetylcholine inhibits dorsal raphe neurons (Koyama and Kayama, 1993), suggesting that there may be a muscarinic component to the cholinergic modulation. An auto-radiographic study, using a pan-muscarinic ligand, reported the presence of muscarinic receptors in the raphe (Cortes et al., 1984), supporting the concept that the cholinergic system may exert opposing effects on the serotoninergic system. Further complexity is added by the finding that approximately 90% of cholinergic neurons in the pedunculopontine and laterodorsal tegmental nuclei express 5-HT_2A_ receptors (Morilak and Ciaranello, 1993) and that serotonin has been shown to inhibit laterodorsal tegmental neurons (Koyama and Kayama, 1993); suggesting a feedback exists between the two systems.

Acetylcholine has been reported to stimulate serotonin release in the caudate via nicotinic (Becquet et al., 1988; Reuben and Clarke, 2000) but not muscarinic (Becquet et al., 1988) receptors. This effect was blocked by a GABA antagonist, indicating this might be an indirect effect, modulated by the GABAergic interneurons (File et al., 2000). Nicotine was shown to stimulate serotonin release in the hippocampus (Kenny et al., 2000) and frontal cortex (Ribeiro et al., 1993), an effect that was inhibited by methyllycaconitine in the hippocampus (Tucci et al., 2003), implicating the α7 nicotinic receptors. Interestingly, the muscarinic antagonist pirenzepine also stimulated hippocampal serotonin release (Kenny et al., 2000), suggesting that M1 or M4 may tonically inhibit hippocampal serotonin release. Together these data support the concept that the cholinergic system can enhance serotonergic activity via nicotinic receptors. Although there are indications that muscarinic receptors have an inhibitory role in serotonergic regulation, more research is required to address this hypothesis.

#### Serotonergic regulation of acetylcholine

The basal forebrain receives afferents from numerous systems, including serotonergic fibers from the dorsal raphe (Semba et al., 1988). An autoradiographic study revealed the presence of predominantly 5-HT_1_ with fewer 5-HT_2_ receptors in the basal forebrain (Zilles et al., 1991), an immunohistochemical study later identified 5-HT_1A_ receptors on the cholinergic neurons (Kia et al., 1996). Serotonin and 5-HT_1A_ agonists have been shown to cause hyperpolarisation of cholinergic cells (Khateb et al., 1993), suggesting that serotonin can regulate basal forebrain cholinergic neurons.

5-HT_1A_ agonists were also shown to facilitate acetylcholine release in the cortex (Bianchi et al., 1990; Katsu, 2001; Millan et al., 2004) and hippocampus (Lazaris et al., 2003; Millan et al., 2004). However, a similar effect is also seen with 5-HT_1A_ antagonists in both cortex (Kehr et al., 2010) and hippocampus (Schechter et al., 2005; Kehr et al., 2010), suggesting that the opposing actions may be mediated by direct and indirect mechanisms, possibly involving interneurons. In addition to the complexity of the 5-HT_1A_ receptor, the autoreceptor, the 5-HT_1B_,_1D_ in guinea pigs and humans (Hoyer and Middlemiss, 1989), is proposed to tonically inhibit cholinergic neurons (Maura et al., 1989; Rutz et al., 2006) and stimulation of the 5-HT_3_ receptors decreased acetylcholine release (Bianchi et al., 1990). Furthermore, activation of the 5-HT_2A_ (Nair and Gudelsky, 2004) and 5-HT_4_ (Johnson et al., 2012) receptors increase cortical acetylcholine release, whilst stimulation of 5-HT_2C_ and 5-HT_7_ receptors were shown to activate striatal cholinergic interneurons (Bonsi et al., 2007). Blockade of the 5-HT_6_ receptors causes increases in acetylcholine release (West et al., 2009), suggesting they may be involved in the tonic inhibition of the cholinergic system. Since cholinergic neurons do not appear to express 5-HT_6_ receptors (Marcos et al., 2006), the mechanism is probably an indirect one, possibly involving glutamatergic neurons. Although serotonin does appear to be capable of modulating the cholinergic system, the effect depends both upon the receptor stimulated and its localization, making the regulation extremely complex.

#### Acetylcholine and serotonin in schizophrenia

The most reproduced finding for the cholinergic system in schizophrenia is a decrease in central muscarinic receptors, in particular the M1, in people with the disorder [see (Scarr and Dean, 2008) for a comprehensive review]. There are indications that M1 and/or M4 may tonically inhibit serotonin release (Cortes et al., 1984; Kenny et al., 2000), thus it is possible the decreases in muscarinic receptors seen in schizophrenia reduce the tonic inhibition of serotonin, resulting in an over activation of the system with the potential to cause decreases in post-synaptic receptors, such as the 5-HT_2A_, which have been reported in schizophrenia (see Dean, 2003) and increases in the pre-synaptic receptors, for example the 5-HT_1D_, which have not been reported (Scarr et al., 2004; Dean et al., 2006).

Another reproducible finding in schizophrenia is decreased expression of nicotinic receptors (Martin-Ruiz et al., 2003; Severance and Yolken, 2008), since these receptors regulate serotonin release in the hippocampus and possibly cortex, a loss of these receptors may result in decreased serotonergic function, resulting in increased post-synaptic receptors and decreased pre-synaptic receptors. Thus, it is possible that the small overall increases reported in cortical 5-HT_1A_ receptors (Tauscher et al., 2002; Gray et al., 2006) are part of the serotonergic response to decreased nicotinic receptors.

The most widely reproduced finding for the serotonergic system in schizophrenia is the decrease in cortical 5-HT_2A_ receptors (see Dean, 2003). Since 5-HT_2A_ receptors modulate acetylcholine release in the cortex, it is possible that a loss would result in decreased cholinergic efflux. Such an event would be expected to cause an increase in postsynaptic receptors such as the muscarinic M1,3,4 and the α7 nicotinic receptors, which is not in keeping with the data on the pathophysiology of schizophrenia (see Scarr and Dean, 2008, 2009). Of the other serotonergic receptors implicated in the regulation of the cholinergic system, the 5-HT_1A_ receptor has been reported to be either increased (Hashimoto et al., 1991; Joyce et al., 1993; Burnet et al., 1997) or unchanged (Dean et al., 1999b; Cruz et al., 2004; Scarr et al., 2004), making it difficult to interpret the data. However, two studies have reported small global increases in cortical 5-HT_1A_ receptors (Tauscher et al., 2002; Gray et al., 2006), which may result in increased acetylcholine efflux and the subsequent downregulation of postsynaptic receptors—an outcome consistent with the pathophysiology of schizophrenia (see Scarr and Dean, 2008, 2009). Cortical 5-HT_7_ receptors have been reported to be decreased in schizophrenia (Dean et al., 2006), if their role in the cortex is similar to that in the striatum, this would be expected to result in a similar pattern to that described for decreased 5-HT_2A_ receptors, increased post-synaptic and decreased pre-synaptic cholinergic receptors, which does not fit with the pathophysiology of schizophrenia.

In summary, although the data that best fits with the current knowledge regarding the pathophysiology of schizophrenia is that reduced levels of muscarinic receptors contribute to a reduced inhibition of the serotonergic system and a subsequent decrease in post-synaptic serotonergic receptors, as seen with 5-HT_2A_ and 5-HT_7_ receptors, this is overly simplistic given that these are not the only post-synaptic serotonergic receptors; the data regarding 5-HT_1A_ receptors in schizophrenia is inconclusive but there are no reports of decreased levels in schizophrenia. Studies that look at all components of both systems in a single cohort are required to reach definitive conclusions about the interactions of the two systems in schizophrenia.

Historically, the focus of the pathophysiology of psychiatric disorders has been on markers of neurotransmission. There is, however, growing data suggesting that molecules traditionally associated with a response to inflammation or infection are abnormally expressed in people with psychiatric disorders (see Potvin et al., 2008, for example). Thus, we finish the exploration of the interactions of the central cholinergic system and their relevance to biological psychiatry with a consideration of these pathways.

## Interactions of the cholinergic system with inflammatory and immune pathways

A significant body of literature suggests there are physiologically relevant interactions between the cholinergic system (neuronal and non-neuronal) and inflammatory/immune pathways in the periphery (Bencherif et al., 2011; Pena et al., 2011; Verbout and Jacoby, 2012). Thus, it seems reasonable to hypothesize that similar interactions may be important in modulating central inflammatory-pathways. This portion of this review therefore focuses on the evidence to support such a hypothesis. However, in considering this data it is important to acknowledge there is a growing body of evidence to suggest that proteins involved in peripheral inflammatory/or immune processes may have more diverse roles in the central nervous system (Dean, 2011). This means that changes in individual proteins, linked to inflammatory or immune processes in the periphery, may not be indicative of a derangement of the same processes centrally.

### Cholinergic modulation of central inflammatory/immune system

The hypothesis that the cholinergic system is involved in modulating central inflammatory and/or immune system is perhaps best tested at the systems level. With this regard, it is significant that microglial cells, which are widely viewed as resident macrophages in the central nervous system, express α7 nicotinic receptors, activation of which attenuates the pro-inflammatory responses in cultured microglial cells (Carnevale et al., 2007). These data appear to be relevant centrally because it has been shown that an α7 agonist, 3-(2,4-dimethoxybenzylidene) anabaseine, reduces tumor necrosis factor (TNF)-α release *in vivo* (Giebelen et al., 2007). Moreover, the relationship between the cholinergic system and, at least cytokines, seems to be a “whole of body” association since the same outcome has been observed in blood after treatment with an α7 agonist (Li et al., 2011). Interestingly, the activity of the α7 nicotinic receptor is reduced by kynurenic acid (Hilmas et al., 2001) via an as yet unknown mechanism. This is significant because kynurenines are components of pro-inflammatory pathways which have been suggested to be involved in the pathophysiology of a number of psychiatric disorders (Schwarcz et al., 2012). Thus, it is possible that α7 nicotinic receptors may be a convergence point for interactions between disparate inflammatory related pathways, providing a common route to inducing some of the symptoms of a number of psychiatric disorders. Whether or not this is proven to be the case, current evidence clearly supports a potential interaction between the central cholinergic system and an inflammatory/immune response as a mechanism involved in maintaining homeostasis within the central nervous system.

Additional evidence suggests that the ability of the cholinergic system to modulate the activity of microglia may be multifaceted; donepezil, a reversible, non-competitive cholinesterase inhibitor, has been shown to attenuate microglial production of nitric oxide and TNF, possibly by inhibiting the canonical inflammatory NF-κB signaling (Hwang et al., 2010). Whilst these data are difficult to interpret because the doses of donepezil used are higher than the therapeutic dose, they do reinforce a functional interaction between acetylcholine and the inflammatory and/or immune system. This interaction appears to be functional because rivastigmine, another cholinesterase inhibitor, has been shown to ameliorate the inflammation induced in experimental autoimmune encephalomyelitis (EAE) (Kawamata and Shimohama, 2011). In this study, it was shown that rivastigmine reduced demyelination, microglia activation, and axonal damage as well as the production of pro-inflammatory cytokines TNF, interferon γ (IFN) and interleukin (IL) −17. A third cholinesterase inhibitor, neostigmine, has similar effects on inflammatory/immune pathways (Tyagi et al., 2010), suggesting that this outcome is a drug class effect. Moreover, two studies showed these effects were abolished by an α7 antagonist (Kawamata and Shimohama, 2011; Tyagi et al., 2010) reinforcing the concept that the primary interaction between the central cholinergic system and microglia revolve around this receptor.

### Inflammatory/immune system control of central cholinergic system

As with all biological systems, there seems to be the potential for a two-way interaction between the inflammatory/immune systems and the nicotinic system. For example, it has been shown that IL-1β and TNF can alter nicotinic receptor sub-unit assembly (Gahring et al., 2005). In particular, these cytokines affect the way in which α4, β2, and β4 sub-units are incorporated into functional receptors; IL-1β enhancing α4/β2 and decreasing α4/β4 containing receptors, whereas TNF promotes α4/β2/β4 sub-unit containing receptors. It has long been known that changes in nicotinic receptor sub-unit assembly has functional consequences (Dingledine et al., 1999), therefore the effects of these cytokines would be expected to have an impact on central cholinergic neurotransmission. This hypothesis has some support as it has been shown in pre-clinical models that targeting nicotinic receptors with drugs that favor receptors of specific sub-unit composition have different therapeutic outcomes (Nirogi et al., 2011).

**Figure 5:**
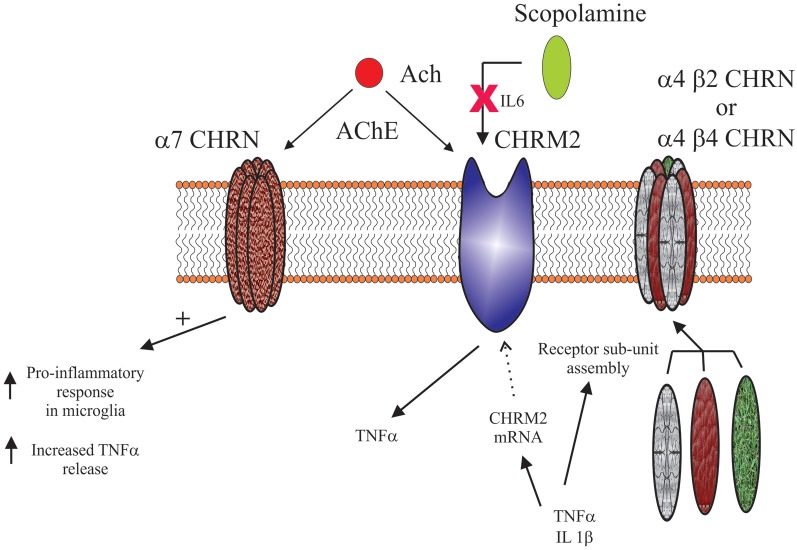
**Schematic showing the complex interactions between cholinergic receptors (α7 nicotinic receptor (α7 CHRN), muscarinic M2 receptor (CHRM2), α4 β2 nicotinic receptor (α4 β2 CHRN) and the α4 β4 nicotinic receptor (α4 β4 CHRN) and the cytokines; tumor necrosis factor α (TNF α), interleukin 1 β (IL1 β) and interleukin 6 (IL6).** Current data suggests some of these interactions may involve cholinesterase inhibitors (AChE) and their ability to regulate acetylcholine (Ach) and the interactions of the muscarinic receptor antagonist scopolamine with CHRM2.

The physiological outcomes of the cholinergic system are usually determined by the balance between nicotinic and muscarinic receptors (Decker and McGaugh, 1991). At present there seems to be little evidence from central studies that such interactions are required in the regulation of inflammatory/immune systems but some data implicates muscarinic receptors as modulators of peripheral interactions (Sales, 2010). Focusing on the muscarinic receptors, the M2 receptor seems to be an important modulator of inflammation/immune pathways in the lung (Costello et al., 1998; Fryer et al., 1999). This proposal is reinforced by the observation that mice which lack M2 receptors have problems with controlling infections and abnormal inflammatory responses (Turner et al., 2010). Significantly, similar to the data for nicotinic receptors, it seems that interactions between M2 and TNF are involved in the cross-talk between cholinergic and inflammatory/immune pathways in the lung (Nie et al., 2011). However, interactions between components of the inflammation/immune pathway and muscarinic receptors appear complex (see Figure 5). For instance, it has been shown that a synergistic action involving TNF and IL-1β reduces M2 expression (Haddad et al., 1996). The interactions between cytokines and muscarinic receptors seem to be quite extensive given the demonstration that IL-6 can reduce the amnesic effects of the muscarinic receptor antagonist, scopolamine (Bianchi et al., 1997). A recent study suggests that interactions between the cholinergic and inflammation/immune system may also involve the M3 receptor (Xu et al., 2012). However, mice lacking M3 have been reported to not have changes in inflammatory/immune responses (Matsui et al., 2000), suggesting a more tenuous link for the receptor in modulating inflammation/immune pathways. Moreover, some of these studies suggest that nicotinic and muscarinic receptors have opposing modulatory roles on the inflammation/immune systems (Razani-Boroujerdi et al., 2008).

### Interactions between the two systems in psychiatric disorders

Somewhat surprisingly, it has been shown that activating central, but not peripheral, M2 receptors modulates levels of TNF in serum (Pavlov et al., 2006), suggesting that these molecules could regulate the interactions between central cholinergic and inflammation/immune systems. This is significant because our laboratory has reported cortical decreases in M2 (Gibbons et al., 2009) and increases in TNF-regulated pathways (Dean et al., 2010, 2012) in people with mood disorders. It is therefore intriguing as to whether these changes are independent of each other or reflect changes in the central cholinergic/inflammation/immune systems. Pursuing this hypothesis would be worthwhile given data showing that muscarinic agonists can reduce TNF levels in rodents (Pavlov and Tracey, 2006) and act as antidepressants (Drevets and Furey, 2010).

Given the clear relationship between the cholinergic and the inflammation/immune systems it remains to conceptualize a mechanism by which this can occur centrally. There are a number of options, one of which is that these changes are the result of interactions between the M2 and α7 receptors and inflammation/immune pathways within microglia. Significantly, it has been shown that carbachol, a pan-muscarinic receptor agonist, caused a rapid influx of calcium into microglia (Zhang et al., 1998), suggesting that they do express functional muscarinic receptors. Although a recent microarray study suggests microglia express both M2 and M3 receptors, it is not known if this is the totality of their muscarinic component (Myers et al., 2009). It has also been reported that microglia, at least in culture, express α7 receptors (Shytle et al., 2004), providing further support for this hypothesis. Given that microglia also produce cytokines (Hanisch, 2002), it is not unreasonable to suggest that the cholinergic/inflammation/immune interactions occur within these cells. However, as cholinesterase inhibitors can also mediate the interaction between the cholinergic and inflammation/immune system (Hwang et al., 2010; Kawamata and Shimohama, 2011) this process appears to be activated by acetylcholine which, in the brain, is likely to be of neuronal origin. Furthermore, M2 and α7 receptors are expressed by neurons (Baghdoyan et al., 1998; Zarei et al., 1999), specifically GABAergic neurons (Azam et al., 2003) and astrocytes (Duffy et al., 2011; Roda et al., 2008). Thus, it is presumptive to assume microglia are the only cells modulating central cholinergic and inflammation/immune interactions. Given the growing recognition that the interactions between these systems may be important in the pathophysiologies of mood disorders, obtaining a better understanding of the mechanisms by which these interactions occur should be a priority.

## Conclusion

To briefly summarise the potential interactions that might occur in psychiatric disorders, in schizophrenia where decreases in M1 are widely reported, these could result in reduced kainate function, which in turn could contribute to a glutamatergic hypofunction. The reduced α7 nicotinic capacity reported to exist in schizophrenia would result in reduced GABA efflux, with the potential to cause increased levels of postsynaptic GABAergic, such as GABA_A_ receptors. An expected consequence of the increased levels of β2 containing nicotinic receptors and the decreased levels of M1 and/or M4 receptors is an increase in dopamine release, potentially contributing to the imbalance in dopaminergic systems proposed to exist in schizophrenia. With regards to the serotonergic system, the decreases in M1/M4 receptors seen in schizophrenia could cause an increase in serotonin release, which would cause the downregulation of postsynaptic receptors, including 5-HT_2A_. Conversely, if the small global increase in the 5-HT_1A_ receptors is substantiated, this could affect the cholinergic system causing increased cholinergic release, a consequence of which might be the downregulation of postsynaptic cholinergic receptors, including the M1 and M4. Finally, it is possible that the dysregulation of molecules traditionally associated with inflammation/immune responses in psychiatric disorders centers around disrupted interactions between the central cholinergic system, mediated by M2 and α7 receptors and microglia.

It is evident from these brief overviews that a dysfunctional central cholinergic system can have far reaching consequences. A common theme in considering these interactions is that the regulatory mechanisms are two-way systems, often with a third implicated as an intermediary. Thus, even considering the interactions between two systems is overly simplistic, suggesting that a whole systems approach is necessary to fully understand the relationships between central systems that become unstable in psychiatric disorders.

## Future directions

In this review, we identified the most commonly replicated changes in neurochemical markers associated with psychiatric disorders and interpreted them in the light of basic research elucidating interactions between the cholinergic and other central neurotransmitter systems. Acetylcholine was chosen as the pivotal transmitter system because of the extent of its innervations and because it is a target of choice for many drug development strategies aimed at novel therapies for psychiatric disorders. For example, acetylcholinesterase inhibitor use has expanded from their initial role of improving cognitive impairment in dementias (Hollander et al., 1986) to their specific use as adjuncts for the treatment of visual hallucinations (Patel et al., 2010; Abad et al., 2011). Efforts to target specific cholinergic receptors to provoke therapeutic outcomes are ongoing, with particular emphasis on the α7 nicotinic (Lieberman et al., 2013) and M1 muscarinic (Patel et al., 2010) receptors to improve cognitive performance. Meanwhile, attempts to develop new antipsychotic agents are focusing on the M4 muscarinic receptor (Leach et al., 2010). In mood disorders, the ability of scopolamine to ameliorate depressive symptoms, in people with major depressive and bipolar disorders (Drevets et al., 2012) has rejuvenated research into new targets for anti-depressant drugs. These developments, combined with the cholinergic regulation of the inflammation/immune system, which appears to play a role in the pathophysiology of psychiatric disorders, made the cholinergic system an obvious choice for the central factor in our review. Increasing our understanding of the interactions between the central neurotransmitter systems will provide alternative means of modulating systems rather than trying to target specific components of the system of interest—which may prove to be undruggable for various reasons. Such an approach has already been used in Parkinson's disease, where anti-cholinergic drugs were employed to ameliorate the tremor associated with the disorder.

One caveat of this review is that most of the data related to the neurochemical changes in psychiatric disorders has arisen from postmortem studies. Therefore, we cannot ascertain which of the chemical changes occurred first, hindering our attempts to construct a theory around these changes. Even if we could look at all of the markers detailed in this review in the same cohort of living people, whilst we would be able to confirm or disprove some of the proposed interactions, we still would not be able to determine the cause and effect relationship.

Furthermore, given the emphasis on the neurodevelopmental aspect of many of these disorders (Sigurdsson et al., 1999; Piper et al., 2012), we do not know at which stage in development such changes occurred. In order to gain a better understanding of the impact disruptions one neurochemical has on others, it would be necessary to model such changes in animals. Ideally, this approach would involve the sophisticated gene knockout techniques that are capable of targeting specific genes in a select group of neurons or tissue (Wess, 2012). Such a course would enable the dysregulation of individual components of neurotransmitter systems at any selected time point during development and allow researchers to assess the effects of disrupting specific interactions at these times. This, in turn would enable the identification of the specific components involved in the interactions between central systems and provide an insight into the long term consequences of specific neurotransmitter system dysfunctions during development.

The development of new technologies and our increasing understanding of the processes involved in the translation from gene sequence to active product also offer a number of new approaches that can be utilized to improve our knowledge regarding the interactions between central transmitter systems. For example, the relatively new field of optogenetics—where light can be used to activate specific neurons—offers great scope to activate specific receptors in tissue of interest and identify the consequences of that activation. This approach will be of particular use in determining which receptors are involved in the cross-talk between transmitter systems, thereby circumventing the problems associated with using drugs that, although they have a high affinity for a particular receptor often have the capacity to stimulate or inhibit the actions of other receptors.

What was once a “simple” process of a gene being transcribed into RNA which was then translated into the corresponding protein is gradually being unraveled to reveal a far more complex series of events than previously imagined. We now know that factors such as gene methylation and histone modification (epigenetics) can determine whether or not a gene can be transcribed. Assuming the RNA is generated, the next step in the process can also be regulated, this time by microRNAs (miRNAs) which have the ability to block the translation of mRNA into proteins. Therefore, these factors also have to be taken into account when considering the interactions between central neurotransmitters, particularly since both epigenetics and miRNAs have been implicated in psychiatric disorders. For example, does the activation of one system affect the prevalence or type of epigenetic markers on the genes that encode components other systems? Will such changes in turn affect the fundamental regulation of expression for that gene? Does transmitter X affect the expression of particular miRNAs? If so, which of the myriad of theoretical interactions ascribed to each miRNA actually occur physiologically and of those, which are relevant to the process under investigation? The involvement of both miRNAs (Dwivedi, 2011; Banigan et al., 2013) and epigenetic markers (Zhao et al., 2012; Sun et al., 2013) in psychiatric disorders mean that a great deal of progress is being made in understanding the consequences of such factors. As screening protocols, such as the miRNA microarray, are developed, they can be applied to the study of interactions between central neurotransmitters, which in turn will feed into our understanding of the neurochemical changes associated with psychiatric disorders, paving the way for the development of targeted therapeutic approaches.

### Conflict of interest statement

Andrew Stuart Gibbons, Madhara Udawela, and Jaclyn Neoreport no competing interests. The following authors have previously received remuneration: Elizabeth Scarrreceived honorarium from Astra-Zeneca and travel support from GlaxoSmithKline (GSK). Brian Deanreceived travel support from GSK, honorarium from Pfizer, Eli Lilly, and Merck Sharp and Dohme (MSD).
